# Long-acting antituberculous therapeutic nanoparticles target macrophage endosomes

**DOI:** 10.1096/fj.14-255786

**Published:** 2016-12-24

**Authors:** Benson J. Edagwa, Dongwei Guo, Pavan Puligujja, Han Chen, JoEllyn McMillan, Xinming Liu, Howard E. Gendelman, Prabagaran Narayanasamy

**Affiliations:** ^*^ Department of Pharmacology and Experimental Neuroscience, and; ^†^ Department of Pharmaceutical Sciences, University of Nebraska Medical Center, Omaha, Nebraska, USA; and; ^‡^ Center for Biotechnology, University of Nebraska–Lincoln, Lincoln, Nebraska, USA

**Keywords:** Mycobacterium tuberculosis, Mycobacterium smegmatis, immunoisolation, MDM, subcellular trafficking

## Abstract

Eradication of Mycobacterium tuberculosis (MTB) infection requires daily administration of combinations of rifampin (RIF), isoniazid [isonicotinylhydrazine (INH)], pyrazinamide, and ethambutol, among other drug therapies. To facilitate and optimize MTB therapeutic selections, a mononuclear phagocyte (MP; monocyte, macrophage, and dendritic cell)-targeted drug delivery strategy was developed. Long-acting nanoformulations of RIF and an INH derivative, pentenyl-INH (INHP), were prepared, and their physicochemical properties were evaluated. This included the evaluation of MP particle uptake and retention, cell viability, and antimicrobial efficacy. Drug levels reached 6 μg/10^6^ cells in human monocyte-derived macrophages (MDMs) for nanoparticle treatments compared with 0.1 μg/10^6^ cells for native drugs. High RIF and INHP levels were retained in MDM for >15 d following nanoparticle loading. Rapid loss of native drugs was observed in cells and culture fluids within 24 h. Antimicrobial activities were determined against Mycobacterium smegmatis (*M. smegmatis).* Coadministration of nanoformulated RIF and INHP provided a 6-fold increase in therapeutic efficacy compared with equivalent concentrations of native drugs. Notably, nanoformulated RIF and INHP were found to be localized in recycling and late MDM endosomal compartments. These were the same compartments that contained the pathogen. Our results demonstrate the potential of antimicrobial nanomedicines to simplify MTB drug regimens.—Edagwa, B. J., Guo, D., Puligujja, P., Chen, H., McMillan, J., Liu, X., Gendelman, H. E., Narayanasamy, P. Long-acting antituberculous therapeutic nanoparticles target macrophage endosomes.

Mycobacterium tuberculosis (MTB) is an immediate public health menace. This is heralded by its ease of transmission, delay in diagnosis, communicability, therapeutic adherence, and resistance. Infection is sped by comorbid states such as nutritional deficiencies and human immunodeficiency virus (HIV) infection (1, 2). Disease morbidity and mortality remain common and significant (3, 4). Indeed, the numbers of infected people worldwide now exceed 14 million (5). The routine use of directly observed antimicrobial therapy while ensuring microbial eradication is cumbersome (6). Source case-patient investigations (7), effective treatment regimens (8, 9), and development of novel drugs (10–13) are certainly of immediate need.

The mechanisms of MTB persistence provide clues toward what is needed to improve treatment and preventive outcomes (14). MTB transmitted by infectious aerosols are ingested and replicate within endosomes of alveolar macrophages (AMs) and then spread the organism to adjoining lymph nodes (15–17). AM phagosomes harbor MTB but fail to eliminate the organism. Rapid cell fusion leads to multinucleated giant cell formation and an inability of the host to affect innate antibactericidal responses (18). Indeed, the mycobacterium manages the endocytic pathway for its own survival (19, 20). Phagosome maturation is prevented by MTB through its abilities to disrupt phagolysosomal fusion events (20–23). Consequently, MTB remains dormant for long time periods, measured in years, during which time it is sequestered in macrophage granulomas (24). Microbial latency is terminated during stressful events such as malnutrition, immune deficiencies, or coinfections with viral, bacterial, or parasitic agents (25, 26). These serve to enhance mycobacterial growth and its inevitable dissemination (27, 28).

Currently available drugs used to treat MTB require long treatment intervals without interruption. This ensures that the mycobacterium is targeted and antimicrobial activities are sustained (29). Others have asked whether elimination of the microbe can be facilitated (30). We reasoned that the use of drug targeting to mononuclear phagocytes could facilitate cellular and subcellular drug delivery to sites of active microbial replication and as such would improve therapeutic outcomes (31, 32). To this end, we designed a drug nanocarrier system of rifampin (RIF) and isoniazid [isonicotinylhydrazine (INH)], two commonly used anti-MTB drugs that would bring them to subcellular sites where the pathogen resides (33, 34). The hydrophilic nature of INH restricts intracellular drug bioavailability, as the drug is poorly encapsulated into polymer-based nanodelivery systems. We posit that this can be overcome by the synthesis of a hydrophobic INH derivative, pentenyl-INH (INHP), which improves nanoencapsulation into nanoparticles (NPs). Here we demonstrate that antituberculous NPs can colocalize in identical subcellular organelles to improve the therapeutic index and drug efficacy. These results were shown using human monocyte-derived macrophages (MDMs) as the target cell for Mycobacterium smegmatis infection. Overall, our results demonstrate that specific drug delivery schemes can improve outcomes for mycobacterial infection and as such have real translational potential for human disease.

## MATERIALS AND METHODS

### Materials

RIF; INH; *trans*-2-pentenal; fluoresceinamine isomer 1; poly(d,l-lactide-coglycolide) acid (PLGA) terminated, lactide:glycolide 50:50; and Float-A-Lyser G2 were purchased from Sigma-Aldrich (St. Louis, MO, USA). Pooled human serum was purchased from Innovative Biologics (Herndon, VA, USA). Macrophage colony stimulating factor (MCSF) was prepared from culture fluids recovered from 5/9m α3–18 cells [CRL-10154; American Type Culture Collection (ATCC), Manassas, VA, USA] cultured in ATCC complete growth medium (35). Rabbit anti-human antibodies to Rab 5, 7, 11, and 14 and Alexa Fluor 488 goat anti-rabbit IgG were purchased from Santa Cruz Biotechnology (Dallas, TX, USA). Protein A/G mix magnetic beads were purchased from Millipore (Billerica, MA, USA).

### Synthesis of INHP

*Trans*-2-pentenal was reacted with INH in ethanol at refluxing temperature for 2 h, followed by concentration to half volume. Ether was added to the mixture and kept overnight for precipitation and then filtered and dried to obtain INHP, as described previously (36). ^1^H-NMR was recorded on a Varian Unity/Inova-500 NB (500 MHz; Varian Medical Systems Inc., Palo Alto, CA, USA). Chemical shifts are reported in parts per million (ppm) downfield from TMS, using residual CDCl_3_ (7.27 ppm) as an internal standard. Data are reported as follows: chemical shift, multiplicity (s, singlet; d, doublet; t, triplet; q, quartet; dd, doublet of doublet; m, multiplet; and br, broad), coupling constants and integration. ^1^H-NMR: δ (ppm) 12.0 (1H, br s), 9.02 (2H, br d, *J*=5), 8.32 (1H, d, *J*=9), 8.03 (2H, br d, *J*=5), 6.56 (2H, m), 2.49 (2H, m), and 1.30 (3H, t, *J*=7.5).

### Preparation and characterization of the nanoformulations

PLGA NPs containing either RIF or INHP were prepared by double emulsification using sonication. Briefly, PLGA was dissolved in HPLC-grade dichloromethane (DCM). The drug was then added to the DCM/PLGA solution and mixed to obtain complete dissolution. This solution was added to 1% polyvinyl alcohol (PVA) cooled in an ice bath and sonicated using an ultrasonic processor (Cole Parmer, Vernon Hills, IL, USA) at 20% amplitude for 10 min. Particle size, polydispersity index (PDI), and surface charge (ζ potential) were determined by dynamic light scattering (DLS) using a Malvern Zetasizer Nano Series Nano-ZS (Malvern Instruments, Inc., Westborough, MA, USA). The suspension was mixed overnight at room temperature to evaporate DCM and then collected after 24 h and centrifuged stepwise to 8000 *g* at 5°C for 20 min. After the supernatant was decanted, the pellet was washed twice in 25 ml of deionized water by centrifugation at 8000 *g* for 20 min. The particle size was determined by DLS, and drug concentrations were determined by reversed-phase high-performance liquid chromatography (HPLC) with UV/Vis detection (37, 38).

### Scanning electron microscopy (SEM)

SEM of the NPs was carried out as described previously (39) using a Hitachi S4700 Field-Emission Scanning Electron Microscope (Hitachi High Technologies America, Inc., Schaumburg, IL, USA).

### Synthesis of fluorescent RIF and INHP NPs

Fluorescein-labeled PLGA NPs were prepared as described previously (40). Briefly, PLGA was dissolved in DCM, followed by the addition of *N*,*N*′-dicyclohexylcarbodiimide and *N*-hydroxysuccinimide (NHS) and stirred overnight at room temperature. The urea byproduct was removed by filtration, and the activated ester was used in the next step without further purification. Fluoresceinamine in DMSO was added to activated NHS ester in DCM and stirred in the dark overnight at room temperature. DCM was then evaporated, and the product was precipitated using distilled water. The dye-labeled polymer was purified by repeated dissolution in acetone and precipitation from ethanol and then lyophilized. Dye-labeled PLGA was combined in a 3:1 ratio with nonlabeled PLGA to manufacture dye-labeled NPs as described above.

### Monocyte isolation and culture

Human monocytes were obtained by leukapheresis from HIV-1, HIV-2, and hepatitis B seronegative donors and purified by countercurrent centrifugal elutriation (41). Monocytes were cultured in Dulbecco's modified Eagle's medium (DMEM) supplemented with 10% heat-inactivated human serum, 1% glutamine, 50 μg/ml gentamicin, 10 μg/ml, and 1000 U/ml MCSF at a cell density of 1 × 10^6^ cells/ml at 37°C in a 5% CO_2_ humidified atmosphere (42). After 7 d, MDMs were used for drug pharmacokinetics and antimicrobial assays.

MDM uptake and retention of NPs and native drugs (NDs) were determined as described previously (43). Briefly, MDMs were incubated with a range of drug concentrations, and cell uptake determined over a 24 h period. Adherent MDMs were washed 3 times with phosphate-buffered saline (PBS) and scraped into 1 ml PBS. Cells were pelleted by centrifugation at 1000 *g* for 8 min at 4°C. The cell pellets were resuspended in 200 μl of HPLC-grade methanol, sonicated, and centrifuged at 20,000 *g* for 10 min at 4°C. The methanol extract was stored at −80°C until drug analysis. For cell drug retention studies, MDMs were exposed to drug for 24 h and washed 3 times with PBS and fresh DMEM without drug was added. MDMs were cultured for an additional 15 d with half medium exchanges every other day. On d 1, 5, 10, and 15 following NP treatment, MDMs were collected and methanol extracts prepared. The cell extract samples were stored at −80°C until drug analysis by HPLC.

### Drug quantitation

INH, INHP, and RIF were quantitated by previously published methods (34, 35). Briefly lyophilized NPs dissolved in methanol or methanol cell extracts were injected (20 μl) in duplicate onto a Waters Breeze HPLC system (Waters, Inc., Milford, MA, USA) equipped with a Waters Symmetry C18 column (250 × 4.6 mm × 5 μm) fitted with a C18 guard cartridge. RIF was eluted using a mobile phase of acetonitrile/26 mM potassium phosphate, pH 2.6 (45:55) at a flow rate of 1.0 ml/min and detected at 254 nm. INH and INHP were eluted using a mobile phase of methanol/7 mM sodium phosphate, pH 3.5 (42.5:57.5) containing 0.05% tetramethylammonium chloride at a flow rate of 1.0 ml/min and detected at 254 nm. Drug levels were quantitated by comparison of peak areas to standard curves of free drugs (0.025–100 μg/ml) in methanol.

### Cytotoxicity

Cell viability was determined by the 3-(4,5-dimethylthiazol-2-yl)-2,5-diphenyltetrazolium bromide (MTT) assay as described previously (39). Briefly, MDMs were treated with either individual drugs or combinations at concentrations of 200, 300, or 400 μM for 24 h. The cells were washed with PBS and MTT solution (5 mg/ml) was added; the cells were incubated for 30 min at 37°C, then washed with PBS. DMSO was added and incubated for 15 min at room temperature. Absorbance at 490 nm was quantitated using a SpectraMax M3 microplate reader (Molecular Devices, Sunnyvale, CA, USA).

### Measurement of antimicrobial activity

After drug treatment for 24 h, MDMs were washed 3 times with PBS and then given fresh medium without drug. At d 1, 5, 10, and 15 following drug treatment, the MDMs were infected with M. smegmatis (multiplicity of infection = 1). Following 1 h of infection, cells were washed with PBS to remove extracellular mycobacteria, and fresh medium was added. After 24 h, the cells were washed twice with PBS and scraped into 1 ml PBS. Both cell extract and media samples were stored at −80°C until analysis for mycobacterial infection. Mycobacterium infection was determined by counting colony-forming units (CFU) as described previously (44). Briefly, the cell suspension was treated with 0.25% sodium dodecyl sulfate (SDS) and diluted 100 times. The diluted samples were plated on 1.5% agar and incubated at 37°C for 3 d, and the number of CFU counted.

### Subcellular particle localization

For confocal imaging, monocytes were cultured on a 4-well Lab-Tek II CC2 chamber slide (Nalge Nunc International, Penfield, NY, USA) at a density of 0.5 × 10^6^ cells/well in the presence of 10% human serum and MCSF for 7 d. The cells were treated with 300 μM of fluorescein-labeled RIF or INHP NPs for 8 h at 37°C, washed 3 times with PBS, fixed with 4% PFA for 30 min, permeabilized, and blocked with 0.1% Triton and 5% bovine serum albumin in PBS and then quenched with 50 mM NH_4_Cl for 15 min. The cells were then washed with 0.1% Triton X-100 and incubated with (1:50) Rab 5, Rab 7, Rab 11, and Rab 14 primary antibodies for 1 h at 37°C. The cells were then washed and incubated with the secondary antibody conjugated to Alexa Fluor 488 for 45 min at 37°C. ProLong Gold antifade reagent with DAPI (Molecular Probes–Life Technologies, Grand Island, NY, USA) was added and slides were cover slipped and imaged with a Zeiss LSM 510 microscope (Carl Zeiss, Inc., Thornwood, NY, USA).

### Immunoisolation of subcellular compartments

Immunoisolation of NP-containing endocytic compartments was performed as described previously (45). Briefly, MDMs (45×10^6^ cells) were treated with 300 μM RIF or INHP nanoformulations for 8 h. MDMs were washed with PBS to remove unincorporated NPs and scraped into homogenization buffer [10 mM HEPES-KOH, pH 7.2; 250 mM sucrose; 1 mM EDTA; and 1 mM Mg(OAc)_2_]. The cells were disrupted by 15 strokes using a Dounce homogenizer. Nuclei and unbroken cells were removed by centrifugation at 400 *g* for 10 min at 4°C. Protein A/G paramagnetic beads (20 μl of slurry) conjugated to Rab 5, Rab 7, Rab 11, or Rab 14 antibodies were incubated with the supernatants for 24 h at 4°C. The endocytic compartments were collected using a magnetic separator (Invitrogen–Life Technologies, Grand Island, NY, USA) and drug content of the compartments was determined by HPLC. Binding specificity was tested by exposing the beads to the cell lysates. For mycobacterium quantification, MDMs were exposed to M. smegmatis alone for 1 h and processed as described for the nanoformulations with modifications. Briefly, after incubation of the endosomal compartments with the beads for 24 h, the beads were separated and the compartments washed with sterile PBS. The compartments were then diluted with sterile PBS containing 10% human serum, sonicated for 2 s, treated with 0.25% SDS, vortexed for 30 s, and directly plated onto 1.5% agar plates. CFUs were counted after 3 d of incubation at 37°C in a 5% CO_2_ humidified atmosphere.

### Statistical analyses

Data analyses were carried out using Prism (GraphPad Software, Inc., La Jolla, CA, USA). Significant differences in cytotoxicity response were determined by 1-way ANOVA followed by Bonferroni's multiple comparisons test.

## RESULTS

### Synthesis and characterization of INHP

**Figure 1.**
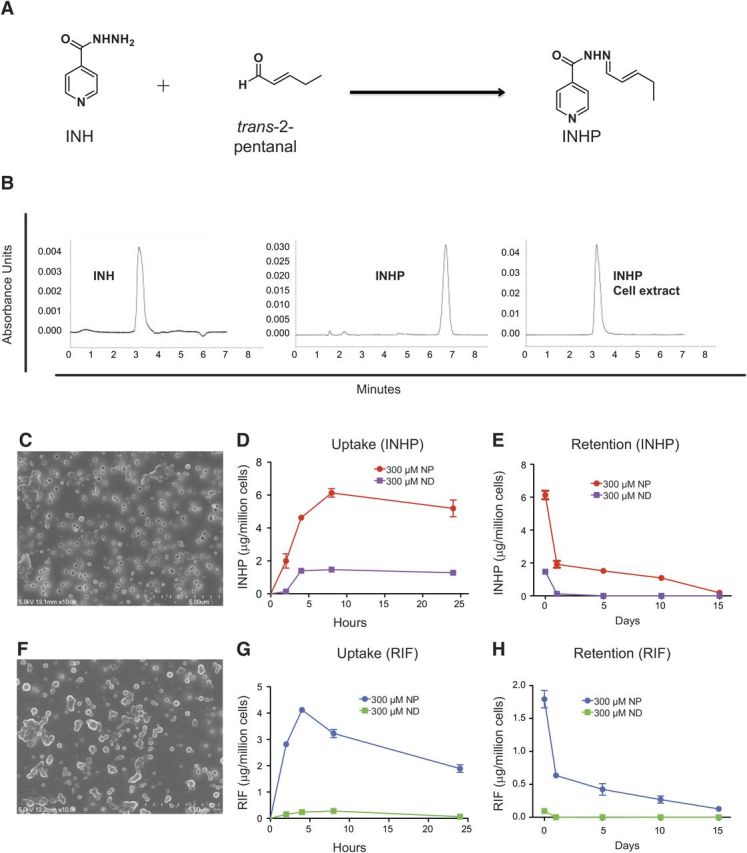
Synthesis of INHP, NP morphology, and comparison between NP and ND uptake and retention in MDM. *A*) Schematic of derivatization of INHP from INH. *B*) HPLC chromatograms of INH, INHP, and INHP after cell extraction. *C*, *F*) SEM images of INHP (*C*) and RIF (*F*) NPs. *D*, *G*) MDM uptake of 300 μM INHP (*D*) or RIF (*G*) NPs or NDs over 24 h. *E*, *H*) MDM retention of INHP (*E*) or RIF (*H*) over 15 d after treatment with 300 μM NPs or NDs. Data for cell uptake and retention are expressed as averages ± sem of *n* = 3 replicates.

INH is a hydrophilic drug, limiting intracellular uptake and encapsulation into PLGA-based particles. This ultimately restricts drug bioavailability, resulting in failure to attain desired therapeutic levels in the body. To improve on nanoencapsulation efficiency of INH, a more hydrophobic derivative, INHP, was prepared by Schiff base formation (**Fig. 1*A***). Analysis of INH and INHP by HPLC demonstrated intracellular cleavage of the derivatizing promoiety to generate the parent drug (Fig. 1*B*). *In vitro* efficacy of INH and INHP NDs against M. smegmatis revealed similar activity for both.

### Characterization of RIF and INHP NPs

PLGA nanoformulations of RIF and INHP were prepared by sonication and characterized by DLS and SEM. RIF- and INHP-PLGA NPs were found to be similar in size and charge. The size and charge of RIF NPs were 219 ± 7 nm and −28.5 ± 3.4 mV, respectively, while INHP particles were 162 ± 3 nm in size with a charge of −23 ± 2.8 mV. Both particles exhibited a narrow PDI (0.11±0.1 for RIF and 0.089±0.01 for INHP particles), indicating uniformity in size. The morphologies of both RIF and INHP NPs were roughly spherical, as determined by SEM (Fig. 1*C*, *F*). Drug loading for RIF and INHP within the PLGA NPs was found to be 10 and 5%, respectively. In contrast, the ND INH achieved a drug loading of ≤1%. At concentrations of 300 μM, both RIF and INHP nanoformulations did not result in cell toxicity as determined by the MTT assay (data not shown).

### MDM uptake and retention of nanoformulated drugs

**Figure 2.**
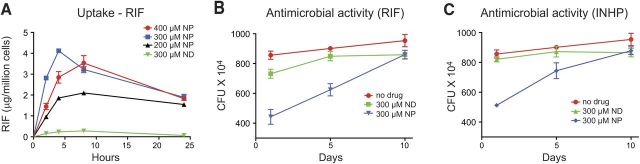
Antimicrobial activity of RIF and INHP NPs. *A*) Concentration-dependent uptake of RIF NP and ND. *B*, *C*) Antimicrobial activity of 300 μM RIF (*B*) and INHP (*C*) NPs and NDs. MDMs were infected with M. smegmatis at d 1, 5, and 10 following drug treatment. Data are expressed as averages ± sem of *n* = 3 replicates.

Uptake and retention of nanoformulated drugs were compared with that of NDs in MDM. As illustrated in Fig. 1*D*, *G*, cell uptake of nanoformulated drugs was 3-fold higher for INHP and 10-fold higher for RIF than uptake of NDs. The nanoformulations were retained in the cells for up to 15 d (Fig. 1*E*, *H*) with drug levels of 0.2 μg/10^6^ cells for INHP and 0.1 μg/10^6^ cells for RIF at d 15; in contrast, the NDs were not detectable after the first 24 h. To determine the RIF concentration that would provide maximum cell uptake, we treated MDM with 200 to 400 μM nanoformulated RIF. As shown in **Fig. 2*A***, treatment with 300 and 400 μM provided similar cell drug levels. At 2, 4, and 8 h, MDM uptake of 300 and 400 μM RIF NPs was 2-fold higher than that for 200 μM.

### Antimicrobial activities of the nanoformulations

Comparison of antimicrobial efficacy of the NDs and nanoformulations was assessed in MDM infected with M. smegmatis. Following a 24 h exposure of MDM to either NDs or nanoformulations of RIF or INHP, cells were infected with M. smegmatis from 1 to 10 d later, and the number of CFUs was determined. NDs demonstrated minimal antimicrobial effects at any time point (Fig. 2*B*, *C*). However, nanoformulations of RIF and INHP exhibited superior antimicrobial activity to NDs at 1 and 5 d after drug loading. The antimicrobial activities of INHP and RIF nanoformulations on d 1 and 5 were 1.6-fold (d 1) and 1.3-fold (d 5) greater than that of the respective NDs. Antimicrobial efficacies of the nanoformulated drugs disappeared by d 10 after drug loading.

**Figure 3.**
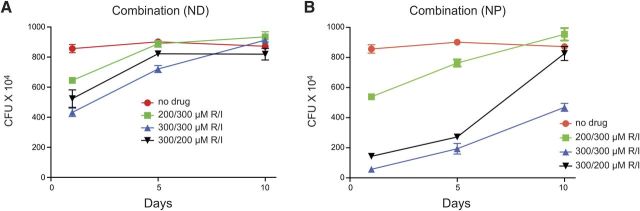
Antimicrobial activity of RIF-INHP combinations. Comparison of antimicrobial efficacy of combinations of NDs (*A*) and nanoformulations (NPs) (*B*) at various concentrations. I, INHP; R, RIF. Data are expressed as averages ± sem of *n* = 3 replicates.

To assess whether combination therapy would improve antimicrobial activity, MDMs were treated with various concentrations of RIF/INHP combinations of either nanoformulations or NDs before M. smegmatis exposure. We evaluated the effect of individual drug concentration on mycobacterium suppression by varying the concentration of each drug used in combination for both NDs and the nanoformulations (**Fig. 3**). Comparison of mycobacterium suppression profiles for the nanoformulations and NDs followed a similar trend. Combined RIF/INHP at 300/300 μM exhibited the most sustained antimicrobial efficacy and enhanced antimicrobial activity for both NDs (Fig. 3*A*) and nanoformulations (Fig. 3*B*) compared with individual drugs (Fig. 2*B*, *C*). Of significance, the 300/300 μM RIF/INHP nanoformulations suppressed mycobacterial replication over a 10 d period. The antimicrobial activity on d 1 and 5 after drug loading for the RIF/INHP nanoformulations was 3- and 5-fold greater than for native RIF/INHP. In the 300/200 μM RIF/INHP treatments the differences in the activity against mycobacterial replication was up to 4-fold higher in the nanoformulation arm when compared with NDs on d 1 and 5 after drug loading. Treatment of MDM with 200/300 μM RIF/INHP resulted in a 2-fold difference in antimicrobial efficacy between the nanoformuations and the NDs. For NDs a concentration of 200 μM of either drug in the combination suppressed mycobacterial infection 1.3- to 1.7-fold on d 1 after drug loading but was diminished by d 5. For nanoformulated drug combinations, a combination of 300/300 μM RIF/INHP provided 4.5- to 5-fold suppression of mycobacterial growth on d 1 and 5 after drug loading, while the 200/300 μM RIF/INHP combination provided only 1.1- to 1.5-fold suppression. Of significance, no mycobacterium suppression was observed for either NDs or the nanoformulations when infection occurred on d 10 after drug loading if the concentration of either drug in the combination was reduced to 200 μM.

### Confocal microscopy

**Figure 4.**
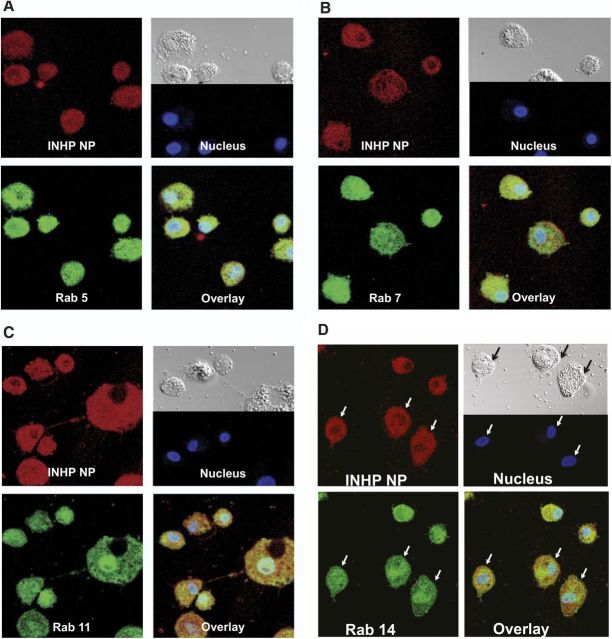
Subcellular localization of INHP NPs. MDMs were treated with 300 μM dye-labeled INHP NPs for 8 h. Cells were fixed with 4% PFA and probed with antibodies to Rab 5 (*A*), Rab 7 (*B*), Rab 11 (*C*), or Rab 14 (*D*). Primary antibodies were detected with Alexa Fluor 488-labeled secondary antibody. Nanoparticles are shown in red, cell compartments in green, nuclei in blue, and overlay of the compartment and particle in yellow.

**Figure 5.**
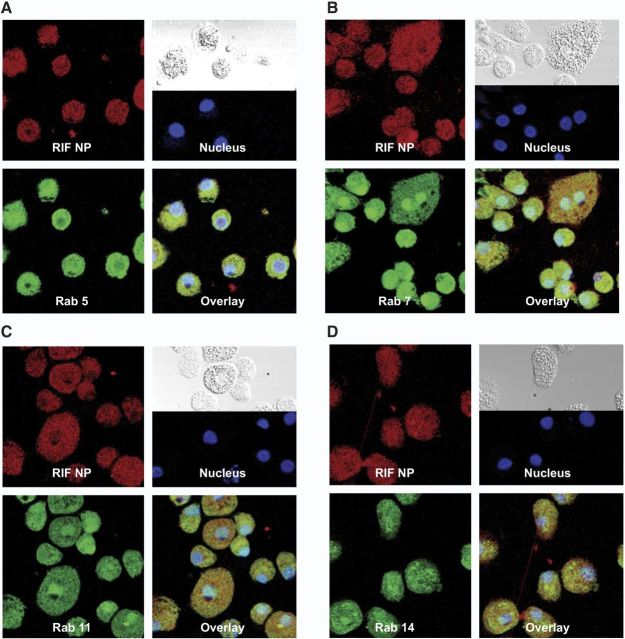
Subcellular localization of RIF NPs. MDMs were treated with 300 μM dye-labeled RIF NPs for 8 h. Cells were fixed with PFA and probed with antibodies to Rab 5 (*A*), Rab 7 (*B*), Rab 11 (*C*), or Rab 14 (*D*). Primary antibodies were detected with Alexa Fluor 488-labeled secondary antibody. Nanoparticles are shown in red, cell compartments in green, nuclei in blue, and overlay of the compartment and particle in yellow.

To determine the subcellular localization of the nanoformulations, MDMs treated with fluorescein-labeled INHP and RIF NPs for 8 h were probed with antibodies to Rab 5 (early), 7 (late), 11 (slow recycling) and 14 (fast recycling) endosomal compartments. Colocalization of NPs and Rab compartments was determined by confocal microscopy. Confocal imaging showed both INHP (**Fig. 4**) and RIF (**Fig. 5**) NP distribution throughout the cytoplasm, colocalizing with late (Rab 7) and recycling (Rab 11 and 14) endosomal compartments.

### Subcellular distributions of NPs and the mycobacterium

**Figure 6.**
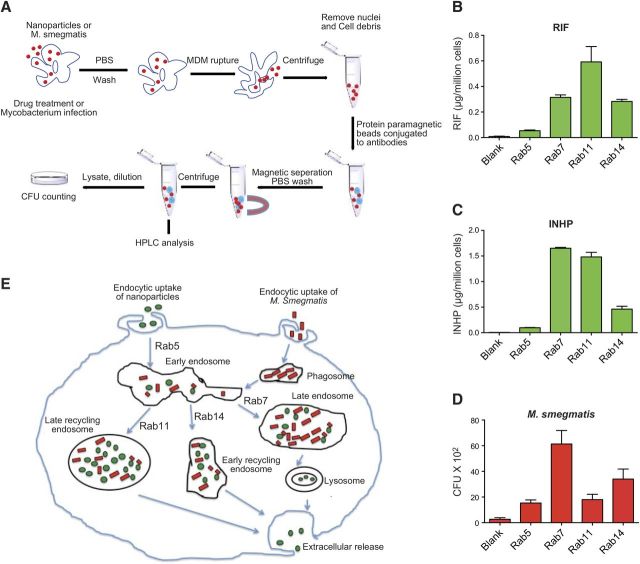
*A*) Schematic diagram of immunoisolation of NPs and M. smegmatis-containing endosomes. MDMs were treated with NPs or M. smegmatis side by side. MDMs were then washed in PBS and ruptured in homogenization buffer. Nuclei and unbroken cells were removed by centrifugation. Protein A/G paramagnetic beads conjugated to antibodies were incubated with the supernatants, and the beads containing endosomal compartments were washed and collected on a magnetic separator. Drug content of each compartment was determined by HPLC after sonication. For mycobacterium quantification, the compartments were diluted with sterile PBS (containing 10% human serum), treated with 0.25% SDS, and loaded onto agar plates for CFU counting. *B–D*) Following immunoisolation, RIF (*B*), INHP (*C*), and M. smegmatis (*D*) were quantitated in subcellular endosomal compartments by HPLC or CFU counting. *E*) Schematic diagram of intracellular pathways of NPs and M. smegmatis. RIF and INHP NPs (shown in green) and M. smegmatis (shown in red) are phagocytosed by MDMs and transported to early endosomes. Nanoparticles and mycobacteria are then either sorted into late endosomes (Rab 7) for release as secretory lysosome or into fast recycling (Rab14) or slow recycling (Rab 11) endosomes for eventual extracellular release.

The trafficking of the nanoformulations through endosomal compartments was compared with subcellular sites of mycobacterial replication. Endosomal compartments from drug-loaded and infected MDMs were immunoisolated using magnetic beads coated with antibodies to Rab 5, 7, 11, and 14 compartments (46). Drug and mycobacterium levels were determined in each compartment. As shown in **Fig. 6**, the RIF and INHP NPs were distributed mainly to late endosomal (Rab 7) and recycling endosomal (Rab 11 and Rab 14) compartments (Fig. 6*B*, *C*, respectively). Similarly, mycobacteria were found in all the endosomal compartments (Fig. 6*D*), with the majority in Rab 7 (late) and Rab 14 (fast recycling) endosomes. These data demonstrate that the drug NPs traffic to the same subcellular compartments where the mycobacterium replicates (Fig. 6*B–D*).

## DISCUSSION

A fundamental limitation in the treatment of MTB is the long duration of therapy required for infection cure. This has been complicated by multidrug-resistant MTB strains unresponsive to traditional therapy. The recalcitrance of MTB to therapy is likely a result of its achieving a dormant state in the host. Since virtually all classes of antibiotics require bacterial replication for their action, the nonreplicating MTB is thought to render it phenotypically resistant to bactericidal antibiotics. MTB drug discovery efforts have been guided by the notion that MTB achieves a latent state as the result of specific interactions with the host, particularly residence in tuberculous granulomas (47). This belief increases the imperative to understand and overcome MTB-specific mechanisms facilitating its dormancy.

Understanding the trafficking pathways of mycobacteria and antimicrobial therapies inside the cell is essential to developing ideal drug delivery systems. This would ensure targeted drug deliveries to mycobacterium reservoirs, reduction in the duration of therapeutic treatment, and a decline in systemic toxicities. Earlier important studies on mycobacterium trafficking pathways reported in literature have focused on the identification of mechanisms by which mycobacteria evade degradation by host macrophages (20, 23, 26, 48, 49). Despite these reports, much is unknown about which endosomes contain the pathogens and the anti-MTB therapy. This formed a sound basis for this work aimed at investigating on the subcellular distribution of the antituberculous NPs and M. smegmatis in MDM for quantification and antimicrobial activities.

At the outset of this work, we manufactured PLGA NPs encapsulating RIF and INHP antituberculous agents. Even though PLGA NPs have previously been shown to exhibit relatively low entrapment efficiency, their small spherical particle size, high surface charge, and low PDI are known to increase bioavailability of many drugs. PLGA has also been extensively used in formulations because of its biocompatibility and biodegradability (50). Formulating RIF is highly desirable to overcome the adverse side effects associated with systemic distribution (43, 51, 52). We manufactured PLGA NPs loaded with RIF by sonication. Drug loading for these NPs was ∼10% and the acceptable particle size of 219 nm was slightly larger than that of INHP NPs, perhaps due to the difference in loading capacities of the nanocarriers. The high payloads would improve the efficiency of drug delivery. When compared with RIF NDs at the same concentration, MDMs demonstrated preferential uptake for nanoformulations as supported by significant enhancement of drug content of over 10-fold, increasing the chances of maintaining therapeutic concentrations inside the cell. We hoped that this could be of advantage during antimicrobial efficacy studies. Similarly, the retention behavior of RIF NPs was characterized by an initial rapid drop in the amount of detectable drug, followed by a slower and sustained decrease over 15 d. In contrast, native RIF was released from MDMs within 24 h.

Having prepared and characterized RIF PLGA NPs, our next goal was to manufacture INHP PLGA NPs. Derivatization of INH to INHP was guided by reported procedures that have demonstrated that hydrophobic analogs of this drug improve nanoencapsulation into various excipients (53). As reported earlier (36), INHP was prepared by derivatization of INH with *trans*-2-pentenal and confirmed by NMR. Antimicrobial response and MTT-based assay of both INH and INHP free drugs indicated that the 2 NDs were of the same activity. Neither of the two drugs elicited cell toxicity at 300 μM concentration. However, this derivatization significantly improved INHP drug loading within the PLGA NPs to 5%. In contrast, encapsulation of the highly hydrophilic INH into PLGA NPs achieved drug payloads of <1%. As reported in literature, this physical interaction could be explained by the lipophilic nature of INH derivatives, characterized by high partition coefficient values of ∼3.2 (36, 54).

SEM images revealed that RIF- and INHP-PLGA NPs are roughly spherical in shape with smooth surfaces. These morphologies have previously been observed for other PLGA NPs. The NPs had a PDI < 0.11, indicating no aggregation of particles. Lack of aggregation was further supported by high negative ζ-potential values that would give rise to charge repulsion between the particles, thereby promoting even distribution. Previous studies in our laboratory have shown that MDM have high preference for particles with a stronger charge (55). High NP uptake by MDM would ensure sufficient drug levels inside the cell where the pathogens replicate.

The size of the manufactured INHP NPs was found to be 162 nm, which makes them ideal for prolonged circulation in the body (55–58). In addition to enhanced uptake by MDM, INHP NPs were retained inside the cell for >15 d. This sustained retention profile would ensure improvements in the frequency of dosing. It should be noted that both INH and INHP are prodrugs. Given that the mode of action and metabolites derived from INH have been contentious since its discovery (59, 60), some of the earlier reports utilized dialysis to evaluate release profiles of the drug from NPs (61). We decided to study uptake and retention kinetics in MDM since dialysis is dictated by particle size, thereby overlooking intracellular processes. After MDM treatment, both INH and INHP displayed the same retention times on analysis by reversed phase HPLC (Fig. 1*B*). The similarity in retention times for the native INH and INHP NPs was only evident following extraction of the drugs from MDM (Fig. 1*B*). It is worth noting that INH was derivatized to INHP through aliphatic Schiff base formation. Aliphatic Schiff bases are known to break easily under slightly acidic or basic conditions; it is therefore possible that the analog reverted to the stable parent INH on extraction from the cell. It is also likely that conversion of INHP to INH could have occurred inside the cell (36).

Uptake of RIF PLGA NPs was evaluated at various drug concentrations to determine the drug concentration that would give maximum uptake without cytotoxicity. MDM uptake of the RIF NPs was maximal at 300 μM. Although uptake and retention kinetics of the nanoformulations was superior to NDs, we could not rationalize this observation to antimicrobial activity since sufficient drug levels are required in the endosomal compartments where mycobacteria grow. We therefore decided to conduct *in vitro* efficacy studies of the nanoformulations against M. smegmatis relative to the NDs.

The concentration dependency of antimicrobial activity was assessed to determine the minimum dose that would give maximum benefit. Additionally, the data generated would give insights on the dosing frequency at various drug concentrations. MDMs were exposed to drug before infection with M. smegmatis, and efficacy was determined 3 d after infection. Antimicrobial activity was improved when INHP and RIF were used in combination rather than individually. It is well established that the most effective TB therapy is comprised of a multidrug combination of INH, RIF, PZA, or ethambutol (8). As shown on Fig. 3, the activity of the 300/300 μM nanoformulation ratio resulted in significantly greater suppression of M. smegmatis when compared with the equivalent concentration ratio of NDs. The enhanced efficacy was most notable when cells were infected 10 d after drug treatment, with NDs exhibiting no antimicrobial efficacy against the fast-growing M. smegmatis, while nanoformulated drugs suppressed mycobacterial growth by ∼50%. These differences can be explained in terms of the ability of the nanoformulations to improve drug uptake and retention by MDM, ensuring that there is sufficient drug concentration over an extended period of time to suppress mycobacterial replication. Antimicrobial efficacy of 300/200 μM or 200/300 μM RIF/INHP against M. smegmatis also demonstrated that the nanoformulations were therapeutically superior to NDs. Of interest the efficacy of the nanoformulated drug combination was greatly increased when the concentration of both drugs was kept at 300 μM. Drug concentration ratios < 200 μM were not effective at inhibiting mycobacterial replication (data not shown). These observations clearly indicate that efficacy against mycobacterial replication is significantly enhanced when nanoformulations of the two frontline drugs are used in combination. RIF and INH have different mechanisms of bactericidal activity, and this could explain the improved activity when both drugs are used together.

For any antimicrobial agent to offer maximum therapeutic benefit, the delivery system should efficiently translocate the drug to intracellular compartments where the pathogens reside and replicate. Delivery and release of antituberculous agents into the granulomas have the potential to subdue MTB, which can survive and multiply within human macrophages. Therefore, understanding how the drug and pathogen interact at the subcellular level forms a platform for better management of TB. To account for the antimicrobial efficacy results, we performed confocal microscopy to evaluate endocytic distribution of the NPs with early (Rab 5), late (Rab 7), and recycling (Rab 11 and Rab 14) endosomes. The NP distribution in all the compartments colocalized in large measure, but not exclusively, with Rab 11 and Rab 7 as compared with early endosomes (Rab 5). These results indicated that the nanoformulations were protected, at least in part, inside recycling endosomal compartments. These observations confirmed that indeed MDM take up and protect the nanoformulations for sustained drug retention. This is reinforced by recent data from our laboratory demonstrating that antiretroviral NPs residing in Rab 7 late endosome compartments can be sustained inside the cells by down-regulation of lysosomes normally designed as clearance mechansims (data not shown). Nanoformulation protection inside the recycling endosome could offer a possible explanation for the observed sustained antimicrobial activity associated with the PLGA nanoformulations. To further substantiate confocal results, we investigated the interaction of the NPs and mycobacteria at the subcellular level in parallel through immunoisolation of the endosomes using protein paramagnetic beads conjugated to Rab5, Rab 7, Rab 11, and Rab 14, following drug treatment or infection with M. smegmatis. The drug content and mycobacteria in each cell compartment were quantified by HPLC analysis and CFU counting, respectively. Consistent with the confocal microscopy data, drug quantification revealed higher drug levels associated with recycling and late endosomes. Interestingly, the subcellular distribution of M. smegmatis was similar to that of drug, with more CFU associated with late endosomes (Rab 7). Interaction of the nanoformulations and mycobacteria in these endosomal compartments can account for the enhanced antimicrobial activity of the nanoformulated drugs. These data provide detailed parallel endocytic trafficking pathways for the nanoformulated antituberculous drugs and mycobacteria. These findings are important for developing formulations to eradicate tuberculosis and other intracellular infections.

## CONCLUSIONS

In summary, novel NPs encasing RIF and INHP antituberculous therapies were successfully synthesized. Our study demonstrates that PLGA NPs encapsulating RIF and INHP, a chemically modified isoniazid derivative, improve drug uptake, retention, and antimicrobial efficacy in MDM when compared with the NDs. These promising in *vitro* results suggest that NP macrophage targeting has great potential to deliver drugs into the subcellular compartments where the pathogens replicate. Our confocal and endocytic trafficking data reveal that M. smegmatis and the drug nanoformulations interact at the subcellular level thereby enhancing the antimicrobial effect. It should therefore be noted that a macrophage-nanocarrier drug delivery approach is a promising system that would improve outcomes in tuberculosis therapy.

## Abbreviations

CFU: colony-forming unit
DCM: dichloromethane
DLS: dynamic light scattering
DMEM: Dulbecco's modified Eagle's medium
HIV: human immunodeficiency virus
INH: isonicotinylhydrazine (isoniazid)
INHP: pentenyl-INH
MCSF: macrophage colony stimulating factor
MDM: monocyte-derived macrophage
MTB: Mycobacterium tuberculosis
MTT: 3-(4,5-dimethylthiazol-2-yl)-2,5-diphenyltetrazolium bromide
ND: native drug
NP: nanoparticle
PBS: phosphate-buffered saline
PDI: polydispersity index
PLGA: poly(d,l-lactide-coglycolide) acid
PVA: polyvinyl alcohol
RIF: rifampin
SDS: sodium dodecyl sulfate
SEM: scanning electron microscopy
